# TRAIL acts synergistically with iron oxide nanocluster-mediated magneto- and photothermia

**DOI:** 10.7150/thno.36320

**Published:** 2019-08-14

**Authors:** Hanene Belkahla, Eva Mazarío, Anouchka Plan Sangnier, John S. Lomas, Tijani Gharbi, Souad Ammar, Olivier Micheau, Claire Wilhelm, Miryana Hémadi

**Affiliations:** 1 Université de Paris, ITODYS, CNRS-UMR 7086, 15 rue J.-A. de Baïf, F-75013 Paris, France.; 2 Nanomedicine, Imagery and Therapeutics, EA 4662, Université de Bourgogne Franche-Comté, UFR Sciences & Techniques, 16 Route de Gray, 25030 Besançon Cedex, France.; 3 Lipides nutrition cancer, INSERM-UMR 1231, Université de Bourgogne Franche-Comté, UFR Science de Santé, 7 Bd Jeanne d'Arc, 21000 Dijon, France.; 4 Laboratoire Matières et Systèmes Complexes, Université de Paris, CNRS-UMR 7057, 10 rue Alice Domon et Léonie Duquet, 75205 Paris Cedex 13, France.

**Keywords:** iron oxide nanoclusters, TRAIL, photothermal therapy, magnetic hyperthermia, apoptosis

## Abstract

Targeting TRAIL (Tumor necrosis factor (TNF)-Related Apoptosis-Inducing Ligand) receptors for cancer therapy remains challenging due to tumor cell resistance and poor preparations of TRAIL or its derivatives. Herein, to optimize its therapeutic use, TRAIL was grafted onto iron oxide nanoclusters (NCs) with the aim of increasing its pro-apoptotic potential through nanoparticle-mediated magnetic hyperthermia (MHT) or photothermia (PT).

**Methods**: The nanovector, NC@TRAIL, was characterized in terms of size, grafting efficiency, and potential for MHT and PT. The therapeutic function was assessed on a TRAIL-resistant breast cancer cell line, MDA-MB-231, wild type (WT) or TRAIL-receptor-deficient (DKO), by combining complementary methylene blue assay and flow cytometry detection of apoptosis and necrosis.

**Results**: Combined with MHT or PT under conditions of “moderate hyperthermia” at low concentrations, NC@TRAIL acts synergistically with the TRAIL receptor to increase the cell death rate beyond what can be explained by the mere global elevation of temperature. In contrast, all results are consistent with the idea that there are hotspots, close to the nanovector and, therefore, to the membrane receptor, which cause disruption of the cell membrane. Furthermore, nanovectors targeting other membrane receptors, unrelated to the TNF superfamily, were also found to cause tumor cell damage upon PT. Indeed, functionalization of NCs by transferrin (NC@Tf) or human serum albumin (NC@HSA) induces tumor cell killing when combined with PT, albeit less efficiently than NC@TRAIL.

**Conclusions**: Given that magnetic nanoparticles can easily be functionalized with molecules or proteins recognizing membrane receptors, these results should pave the way to original remote-controlled antitumoral targeted thermal therapies.

## Introduction

The application of nanoparticles (NPs) to medicine 1-3 is expected to improve the detection and treatment of many diseases, such as cancers and genetic disorders 4. Magnetic NPs are particularly appealing candidates for diagnosis (MRI) 5, drug delivery 6 and thermal treatment by magnetic hyperthermia (MHT), through the application of alternating magnetic fields (AMF), and photothermia (PT), by exposure to laser radiation 7-10, which are being increasingly used in the field of cancer 11-14. One remarkable advantage of iron oxide NPs is their capacity to induce heating by both MHT and PT processes 15-18. Their biocompatibility provided by the iron natural storage pathway after magnetic NPs degradation is another asset for clinical applications 19.

Three categories of thermal therapies have emerged, depending on the temperature increment 20: (1) “Diathermia treatments” 21 correspond to operating temperatures between 37 and 41 °C. This category is generally combined with chemo- or radiotherapy and is known to induce physiological changes, such as variations of the pH, blood flow and oxygenation, which may improve drug delivery to cells resistant to chemotherapy. (2) “Hyperthermia treatments” 22 where the heating process raises the temperature to between 41 and 48 °C. This causes protein denaturation (unfolding and aggregation of proteins) and also temporary cell inactivation 17. This treatment can be further subcategorized into “moderate hyperthermia” when the temperature is in the 41-45 °C range 23, and “suppressive hyperthermia” for temperatures between 45 and 48 °C, where cell death is mediated by rapid necrosis. The last category (3), called “irreversible injury” 24, refers to a tumor temperature range between 48 and 60 °C, which provokes DNA damage and denaturation, as well as severe and irreversible denaturation of proteins 25.

Whatever the thermal therapy and modality (MHT or PT), iron oxide NPs can be vectorized by functionalization with proteins 18, 26, 27, recognized by specific receptors expressed by the tumor cells. This focuses the heating on cancerous tissues, therefore sparing healthy cells from undesirable side-effects 28, 29. These nanovectors are internalized and act actively or passively to eliminate the target cells. One promising protein vector is the tumor necrosis factor (TNF)-related ligand (TRAIL), a type 2 transmembrane protein 30, which is able to trigger tumor cell death 31, 32 through apoptosis 33. To this end, TRAIL binds to either of its two death receptor agonists 4 and 5 (DR4 or TRAIL 1 and DR5 or TRAIL 2), though engagement of apoptosis has recently been found to proceed mostly through DR4 34. Mechanistically, TRAIL binding to its death receptor agonists leads to the recruitment of the Fas-associated death domain (FADD) causing cell death through the caspase-8-dependent apoptotic pathway 35-37. TRAIL-induced apoptosis efficacy is tightly controlled by the glycosylation status of DR4 38, 39 and DR5 40 41, as well as by the multimerization of TRAIL 42-44. In a recent review 30, the potentiality of the use of TRAIL as a promising anticancer agent was described. It also discussed work on cancer therapy using the grafting of TRAIL onto nanoparticles, such as nanocomposites based on alginate 45, carbon nanotubes 46, and other kinds of inorganic 47 and organic 48 nanoparticles.

Since the death receptor agonists are often overexpressed in cancer cells 49, 50, combining the effects of TRAIL with local NP-mediated hyperthermia, by using TRAIL-functionalized iron oxide nanovectors, appears as a yet-unexplored and appealing strategy. As shown recently, both the functionalization of nanomaterials by TRAIL 43, 46, 51 and its use under moderate global hyperthermal conditions 52-54 enhance TRAIL-induced apoptosis.

With a view to its application in remote-controlled moderate hyperthermia (41-43 °C), TRAIL was grafted onto clusters of iron oxide nanoparticles, further referred to as nanoclusters (NCs), and the ability of this original nanovector to induce selective apoptosis under MHT or PT was evaluated using *isogenic* TRAIL*-resistant breast tumor cells,* proficient or deficient for TRAIL receptors. Comparison of NC@TRAIL-initiated heating by MHT or PT with global heating establishes that the nanovector acts in synergy with moderate hyperthermia. The cell death mechanism was depicted by coupling different techniques (transmission electron microscopy in cells, flow cytometry and fluorescence microscopy). Altogether, it evidenced an apoptotic pathway, composed of mixed late apoptosis and necrosis in some cases, which result from the creation of hotspots in the vicinity of the nanoclusters, leading to an enhancement of the thermal fluctuations which in turn destabilize the membrane and provoke its disruption.

## Methods

*Synthesis, functionalization and characteriza tion of NC and preparation of nanovectors***:** Iron oxide nanoclusters (NCs) 100 nm in diameter were synthesized by the polyol method 55. They were prepared as published elsewhere 56 by using an appropriate amount of iron(II) lactate (4.5 g) as metal precursor, 0.025 mol of distilled water and 250 mL of diethylene glycol (DEG) as solvent. The mixture was refluxed, and the temperature was increased at a rate of 6 °C per min. with mechanical stirring up to the boiling point (230 °C) and maintained at this temperature for 3 h. The mixture was then cooled to room temperature, after which the black dispersion of magnetite Fe_3_O_4_ was washed several times with acetone and ethanol and centrifuged for 15 min at 8000 rpm. The black powder was then dried at 50 °C for several hours.

NCs were then functionalized with (3-aminopropyl)triethoxysilane (APTES) to put amino groups onto their surface 57. The number of amino groups was estimated 56 by colorimetric titration after reaction with the Kaiser reagent (ninhydrin) which forms a Ruhemann blue complex in the presence of amino groups 57. Finally, TRAIL was grafted onto NCs by the formation of an amide bond between the amino groups and the carboxylic acid groups of TRAIL, HSA or Tf which were previously activated with N-hydroxysuccinamide (NHS) and 1-ethyl-3-(3-dimethylaminopropyl)carbodiamide (EDC).

*Instrument:* X-ray photoelectron spectroscopy was carried out to confirm the grafting of TRAIL onto the surface of the NCs (Figure S1A, B). The weight loss was determined by thermogravimetric analysis (Labsys-Evo equipment) for NC@APTES and NC@TRAIL (Figure S1C, D). From this weight loss, the mean number of TRAIL molecules present in NC@TRAIL is estimated to be about 6000 56. The grafting was also checked by comparing the FTIR spectrum of the NC@TRAIL with that of bare NCs, using the KBr method on a Nicolet Magna-IR 860 spectrophotometer. The signature of amide groups is clearly evidenced in the 1650-1550 cm^-1^ range (Figure S1E). Zeta potential and Dynamic Light Scattering (DLS) were measured using a Malvern Nano Zetasizer. The surface charge (ζ) of NC, NC@APTES and NC@TRAIL was determined at room temperature by varying the pH (Figure S1F). The diameter of NC@TRAIL was determined in PBS and in DMEM at 10% and 55% fetal calf serum (FCS) (Figure S1G).

Routine magnetometry was carried out on a Quantum Design MPMS-XL SQUID magnetometer, and magnetization curves were recorded at 5 K (Figure S1H) and 310 K as a function of the magnetic field H at -70 to 70 kOe; the decrease in the saturation magnetization of the nanovectors is attributed to their diamagnetic protein contents. The size and shape of the NCs were analyzed on a JEOL-100 CX Transmission Electron Microscope (TEM) operating at 100 kV. The samples were prepared at room temperature by slowly evaporating a drop of NCs dispersed in ethanol on an amorphous carbon-coated copper grid.

*Cell lines and cell culture***:** The MDA-MB-231 breast carcinoma cell line was purchased from ATCC. TRAIL-receptor-deficient MDA-MB-231 (DKO) cells were generated using the TALEN approach as described by Dufour et al. 34. Figure S10 shows by flow cytometry and western blots that the DKO cell line is deficient in TRAIL receptors DR4 and DR5. MDA-MB-231-WT (wild type) and DKO tumor cells were maintained in 10% FCS-supplemented Dulbecco's modified Eagle's medium (DMEM) containing 4.5% glucose and 2 mM glutamine (Lonza, France). The cells were cultured in 5% CO_2_. Adherent cells were seeded at 30% confluence (approx. 80,000 cells per cm^2^). Pharmacological modulation was initiated at 60-70% confluence. Cells were recovered after stimulation by trypsinization and centrifugation at 500 rpm for 3 min. at 4 °C.

* Photothermal and magnetothermal treatments:* 2.10^5^ MDA cells were incubated in 24-well plates. Cells in each well were trypsinated and centrifuged at 500 rpm for 3 min. They were then treated with a precise concentration of TRAIL, raw NCs or a nanovector. A 10 nM concentration of TRAIL is equivalent to a 4 mM concentration of iron in a nanovector. Dispersions with a final volume of 100 µL in a 500 µL Eppendorf tube were used for both photothermia and hyperthermia measurements. Magnetic heating was performed on a DM3 applicator (nanoScale Biomagnetics) at 471 kHz and a field H = 18 mT for 8 min. Photothermia was induced during 8 min. by a continuous NIR laser at 808 nm (LASER Components S.A.S., France). The sample at 37 °C was illuminated by a 1 cm^2^ laser spot at 0.3 W.cm^-2^, with the laser 4 cm from the sample. To maintain sterile conditions a cover-slip rinsed with ethanol was used when the sample was open. For all the measurements, the temperature elevation was recorded in real time with an infrared thermal imaging camera (FLIR SC7000), and the specific loss power (SLP) was measured from the slope over the first 30 s of heating (first 30 points)^7^.

*Cell viability*: Viability of MDA cells after photothermal treatment was assessed by methylene blue assay. After 8 min. of laser irradiation and 16 h of incubation, cells were washed in cold PBS buffer solution and fixed by methanol for 20 min. at room temperature, washed three times in PBS and stained with 5% methylene blue for 30 min. After three subsequent gentle washes in PBS, methylene blue was eluted in 1% HCl for 4 h at ambient temperature. Optical density (OD) was then measured at 630 nm by a spectrophotometer. Images of the treated cells were acquired on an optical microscope connected to a digital camera.

*Iron quantification by inductively coupled plasma*: In order to quantify iron in the different samples of NCs and nanovectors, they were first degraded in 100 µl of 69% nitric acid (HNO_3_). Several dilutions of these samples were then prepared in 2% HNO_3_ to obtain a final iron concentration in the 1-200 ppb (µg/L) range. Inductively Coupled Plasma-Optical emission spectrometry (ICP-OES) was performed using a SPECTRO ACROS spectrometer.

*Transmission Electron Microscopy (TEM) of cells after treatments*: Right after treatment, cells were fixed with glutaraldehyde (2%) in 0.1 mM sodium cacodylate buffer at room temperature for 1 h. Post fixation included then: 1) contrasting with Oolong Tea Extract (OTE) 0.5%, 2) staining with 1% osmium tetroxide and 1.5% potassium cyanoferrate, 3) dehydration with graded solutions of ethanol, 4) impregnation with hexamethylphosphoramide, and 5) embedding in EPON resin supplemented with benzyldimethylamine (3%), and 6) thin sectioning (70 nm). Imaging of the sections was then achieved with a Hitachi HT 7700 microscope operating at 80 keV.

*Flow cytometry for Annexin V and Propidium Iodide detection***:** 24 h after treatment, cells were labelled using the APC Annexin V Apoptosis Detection Kit with propidium iodide (Biolegend). Cells were detached (100,000 cells per condition), washed twice in PBS buffer, suspended in 100 µl of binding buffer to which annexin V (5 μl) and propidium iodide (PI, 10 μl) were added. After 15 min. of incubation, 400 µl of binding buffer were added, and cells were analyzed with a Cyan ADP 9C flow cytometer (Beckman Coulter, Imagoseine platform, Institute Jacques Monod, Paris). Each analysis is based on a minimum of 50,000 events. For each condition, three analyses on three independent cell samples were performed. Besides, the whole experiment was performed twice. The gating for annexin V and propidium iodide was established each time on the control samples. The threshold for propidium iodide was identical for both experiments (300), while for annexin V it differed between the first (250) and second (1500) experiments. The percent of necrotic and apoptotic cells was calculated for the cell population above this threshold.

*Statistical analysis*: For each condition, the number of independent measurements was systematically greater than 3 (n > 3). Values were expressed as the mean ± standard error of the mean. Significance was assessed using one-way ANOVA. For all values, a minimum of 95% confidence level was considered significant, with *** meaning P < 0.01.

## Results and Discussion

### Magnetic properties of NC@TRAIL and Magnetic Hyperthermia (MHT)

**Figure 1:**
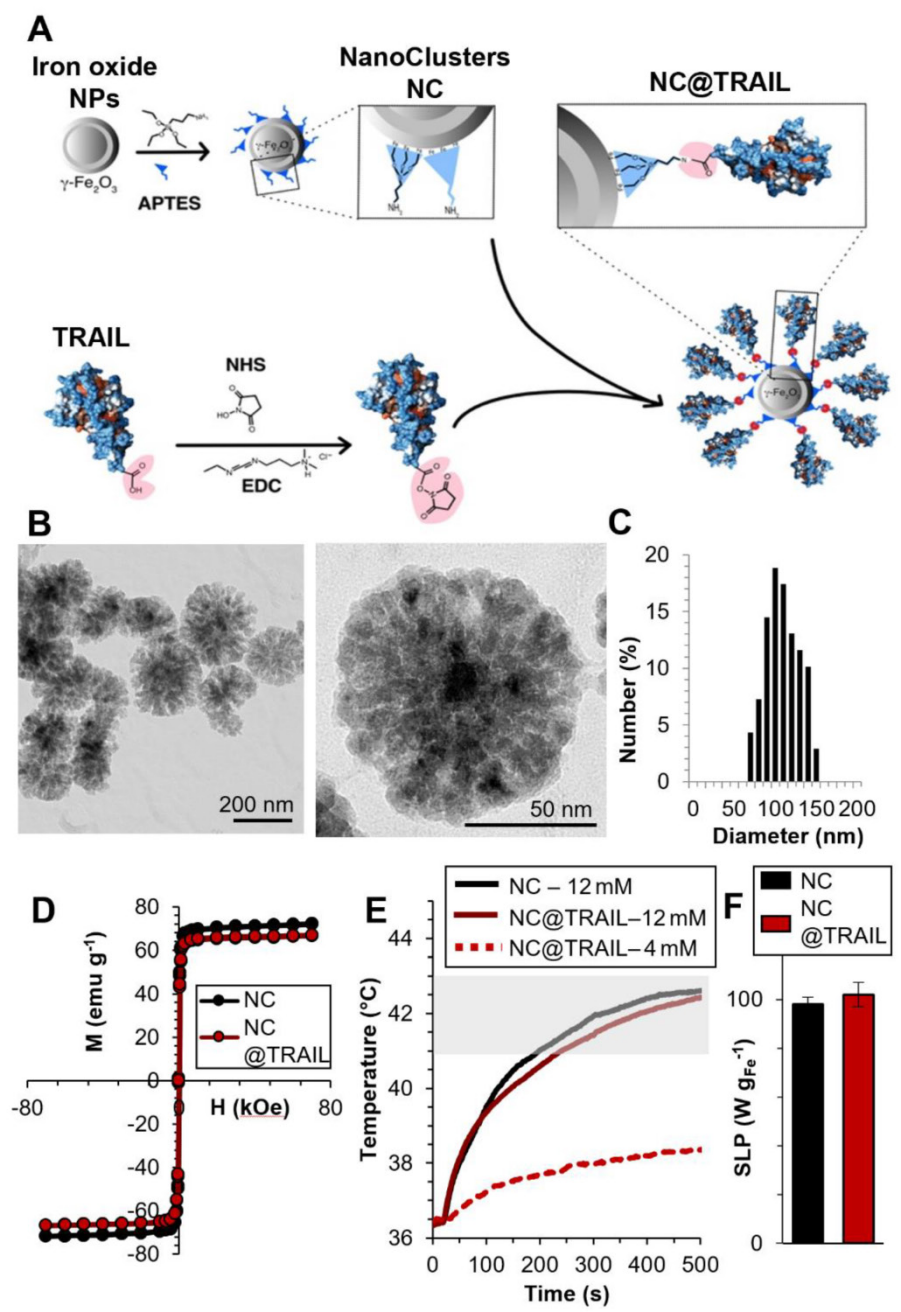
A) Schematic representation of the functionalization of 100 nm iron oxide nanoclusters (NCs) by APTES and the grafting of TRAIL onto NCs via an amide bond between the amino functions of APTES and the previously ester-activated carboxylic groups of TRAIL. B) Transmission Electron Microscopy (TEM) images of iron oxide NCs. C) Size distribution as inferred from statistical analysis using ImageJ software, <D> = 104 ± 20 nm. D) First magnetization curves of NCs and NC@TRAIL (at 310 K). E) Heating curves (temperature versus time) of NCs and NC@TRAIL under MHT for two iron concentrations (4 and 12 mM). F) Heating capacity (SLP in W.g^-1^) of NCs and NC@TRAIL in MHT mode.

TRAIL was grafted onto the surface of 100 nm-diameter iron oxide NCs previously functionalized by (3-aminopropyl)triethoxysilane (APTES) to obtain a TRAIL-targeted iron oxide nanovector, NC@TRAIL (Figure 1A, B, C). NC@TRAIL was characterized by several techniques 56 to highlight the grafting and to determine the number of protein molecules per NC (Figure S1): According to the XPS analyses, the ratio of N/Fe is higher for NC@TRAIL than NC@APTES and NC alone, which indicates the presence of proteins rich in nitrogen (Figure S1A, B). From the TGA, the mass of protein grafted onto NC is about 13% w/w and corresponding to 8000 TRAIL per NC 56 (Figure S1D). The FTIR spectrum displays amide I and II bands, indicating the presence of TRAIL at the surface of the NCs (Figure S1C). The surface charge (zeta potential, ξ) of -15 mV on NC@TRAIL indicates that it is slightly more stable than NC and NC@APTES (Figure S1E). Dynamic diameters were measured by DLS in different media (PBS, DMEM with 10% and 55% FCS, Figure S1F). In PBS the hydrodynamic diameter was 150 nm for NCs, and 140 nm for NC@TRAIL, which indicates better stability of the nanovector. The diameter of NC@TRAIL in DMEM increased to 160 and 210 nm with 10 and 55% FCS, respectively. Such an increase is too small to indicate NC aggregation, and reflects the absorption of serum proteins, especially at high serum concentration.

The magnetic properties were investigated by magnetometry (Figures 1D, S1G). The variation of the magnetization was measured as a function of the magnetic field between -70 and +70 kOe, at 300 K. The saturation mass magnetizations (M_sat_, in emu per g of Fe) of a fixed mass of NC@TRAIL and NC are similar at (66 ± 4) and (71 ± 5) emu.g^-1^, respectively, indicating that the functionalization of NC by TRAIL only slightly reduces its magnetization. Both exhibit superparamagnetic behavior with high saturation mass magnetization (Figure 1D). Magnetization at 5 K is also shown in supplementary Figure S1H, evidencing a small hysteresis feature.

Magnetic heating arises from the energy dissipated in the relaxation of the magnetic moments when NPs are submitted to an alternating magnetic field (AMF). In multi-domain NPs this mechanism is characterized essentially by Brownian motion, which depends on the viscosity of the solvent and the hydrodynamic radius of the NPs 58. MHT measurements were performed using a DM3 applicator (471 kHz, 180 G). Because the magnetic properties of NCs and NC@TRAIL are similar, their heating efficacies under MHT were nearly identical (Figure 1E). Applying an AMF for 8 min. at low iron concentration (4 mM) resulted in a negligible temperature elevation (plateau at 38 °C), but at higher concentration (12 mM), for the same duration, the temperature reached a stable plateau (41-43 °C) in the moderate hyperthermia range (Figure 1E).

Heating efficiency in magnetic hyperthermia is classically expressed as the specific absorption rate (SAR) or specific loss power (SLP), defined by the following equation (1):


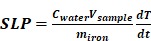
 (1)

where dT/dt is the initial temperature gradient over the first 30 s (first 30 points) after the laser is turned on. C_water_ is the specific heat capacity of water: 4185 J.L^-1^.K^-1^, V_sample_ is the sample volume in L, and m_iron_ is the total mass of iron in the sample in g. Similar SAR values of (98 ± 3) W.g^-1^ and (102 ± 5) W.g^-1^ are found for NC@TRAIL and NCs, respectively (Figure 1F).

### Enhancing TRAIL-induced cell death by magnetic hyperthermia (MHT)

The superiority of NC@TRAIL as compared to TRAIL at 37 °C has been reported elsewhere for HCT116 and HepG2 cancer cells 56. We showed recently that NC@TRAIL is almost 2-fold more potent than TRAIL alone in inducing apoptosis, as monitored by annexin V staining followed by flow cytometry 56. Importantly, this gain of function is not associated with a loss of selectivity since, like the void iron oxide NCs alone and even at high concentration, NC@TRAIL is safe toward normal primary hepatocytes or TRAIL-receptor-deficient HCT116 cells 56. Therefore, the effect of TRAIL aggregation on the nanocluster surface triggers an enhanced cytotoxic response, making NC@TRAIL already better suited as an anti-cancer agent than free TRAIL alone.

**Figure 2:**
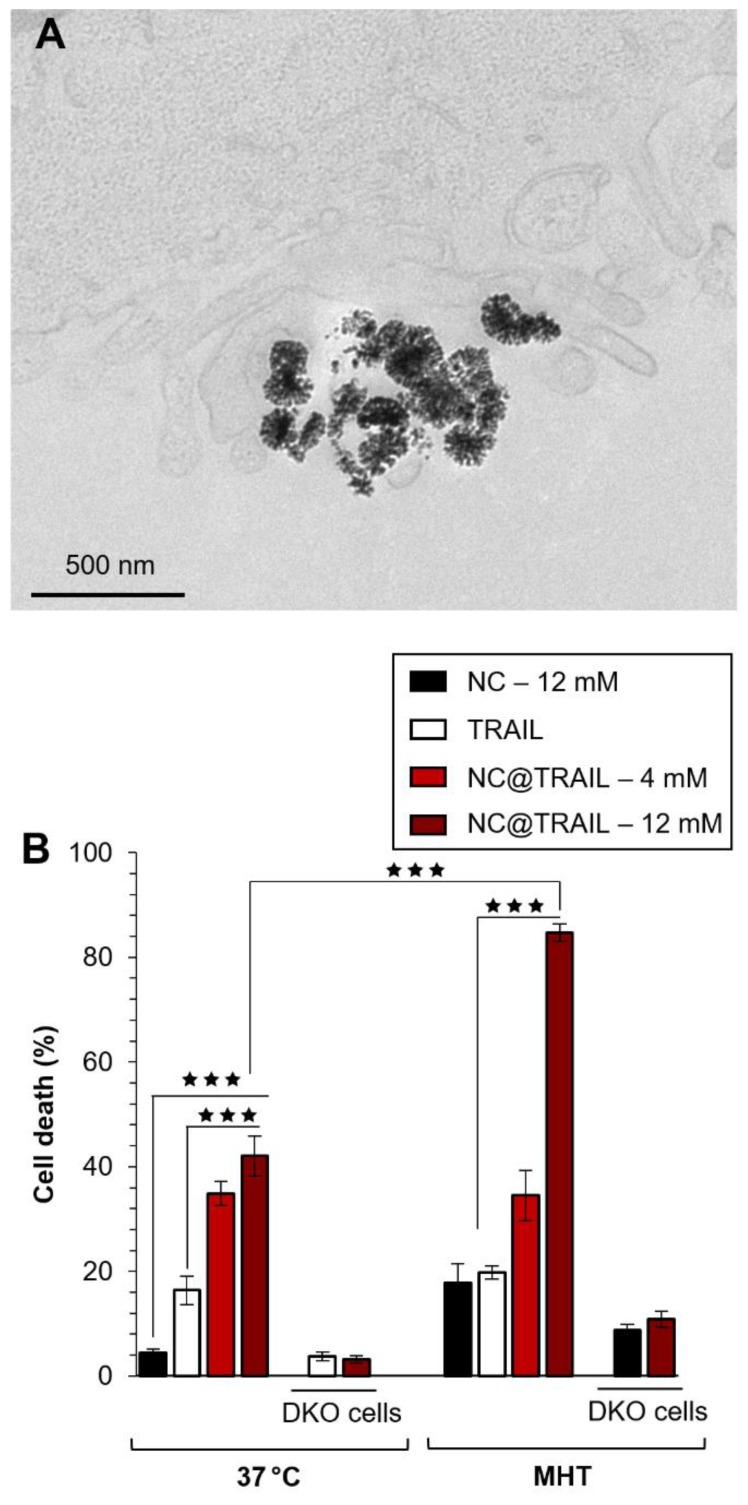
A) TEM image of MDA-MB-231-WT cells incubated with NC@TRAIL at 37 °C; the nanovector is located at the cell membrane. B) Death rate of MDA-MB-231-WT and DKO cells (10% FCS) after incubation with NCs, TRAIL or NC@TRAIL and MHT treatment (right), compared to controls incubated with NCs, TRAIL and NC@TRAIL at 37 °C (left).

For the MDA-MB-231-WT breast cancer cell line, transmission electron microscopy (TEM) was performed on cells incubated with NC@TRAIL at 37 °C (Figure 2A). This Figure demonstrates unambiguously the location of NC@TRAIL at the cell membrane. The cell death rate induced was then determined at 37 °C; Figure 2B shows that it was 20% for TRAIL alone ([TRAIL] = 30 nM), and below 5% for NCs ([Fe] = 12 mM). For NC@TRAIL, it increased to 35 and 42% depending on the iron concentration, at [Fe] = 4 and 12 mM, respectively.

The effect of MHT treatment on the MDA-MB-231-WT cell death rate induced by NCs and NC@TRAIL was next evaluated (Figure 2B, MHT). At an iron concentration of 4 mM, MHT has no impact on cytotoxicity, with the same efficiency for NC@TRAIL with or without MHT, in accordance with the low heating generated at this concentration. The death rate induced by NCs under MHT at 12 mM iron is only 16%, which reflects the therapeutic efficiency of MHT alone. In contrast, NC@TRAIL at the same concentration under MHT resulted in a death rate exceeding 80%, significantly greater than without MHT (42%). This synergy clearly indicates the implication of the TRAIL agonist receptors, DR4 and DR5. The results thus reveal an important synergy of the pro-apoptotic signaling capabilities of TRAIL and moderate MHT. To further confirm this synergistic role, we next performed the same series of experiments, with 12 mM iron, on an isogenic cell line, MDA-MB-231-DKO 34, derived from MDA-MB-231 cells but devoid of TRAIL agonist receptors. Heating induced by MHT is similar for both cell lines (see Figure S2 for heating curves), but Figure 2B (MHT, DKO) clearly shows that the DKO cells respond to MHT with NC@TRAIL exactly as with NCs, with a death rate lower than 5%. All these results are consistent with our previous finding that moderate hyperthermia can enhance TRAIL-induced apoptosis 52.

### Enhancing TRAIL-induced cell death by photothermia (PT)

Photothermal therapy (PTT) is a minimally invasive therapy in which light (photon energy) is converted into heat to induce cell death. It is extensively used with gold NPs because of their unique photothermal effect due to surface plasmon resonance and strong optical absorption 8, 20, 59. Iron oxide NPs were recently found to be very effective mediators in photothermal applications 8, 18, 27, 60, which are competitive with magnetic hyperthermia.

To explore the potentiality of NC@TRAIL for photothermia in the first biological safety window, the molar extinction coefficient was measured at 808 nm: ε = (475 ± 50) M^-1^.cm^-1^ (Figure S3 A,B). The phothothermal conversation efficiency (η) was then calculated from the heating curves, and found to be (23 ± 3)% (Figure S3 C,D). This value of η is high enough to justify the use of such particles for photothermal therapy 61.

**Figure 3:**
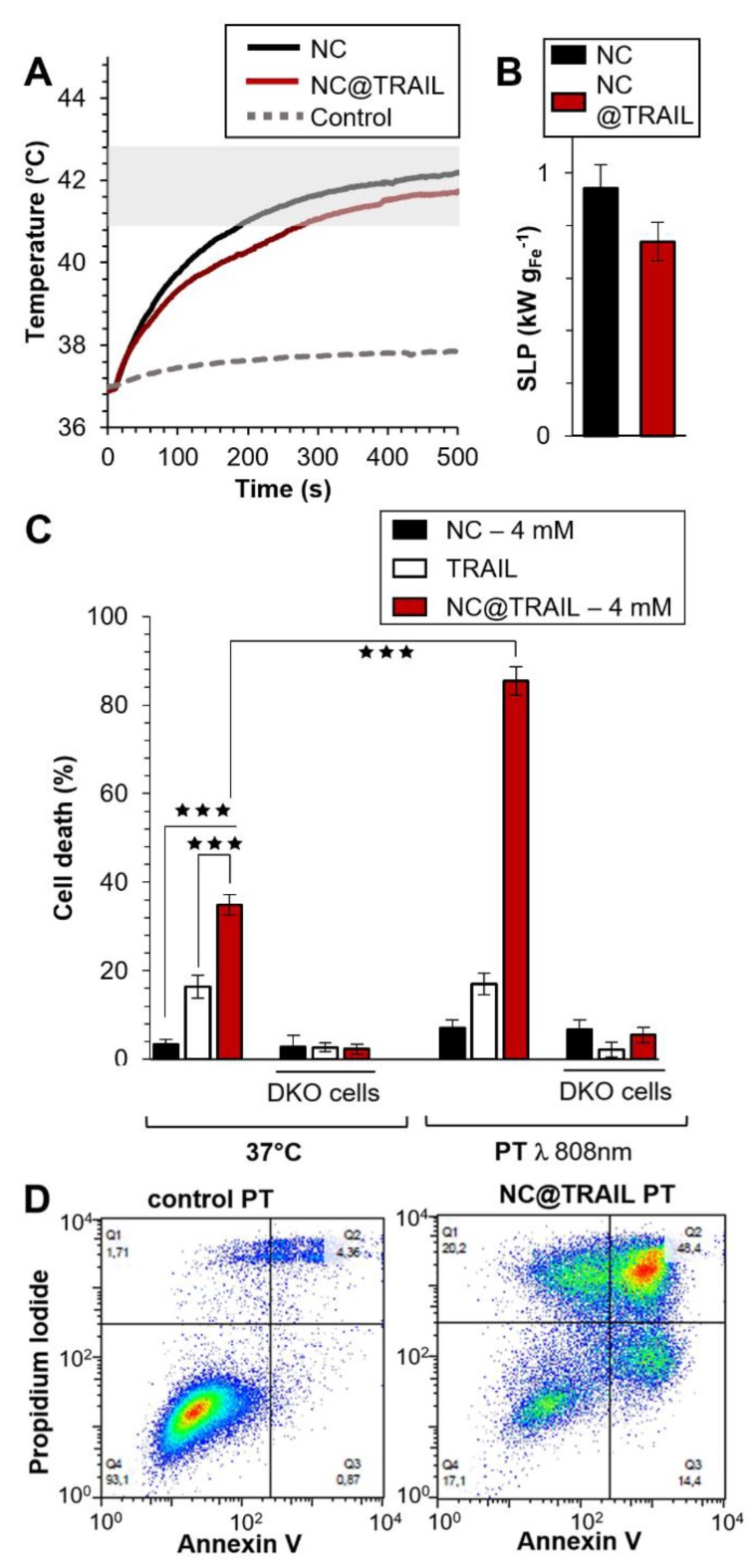
A) Temperature increment for NC@TRAIL (red) and NCs (black) with MDA-MB-231-WT cells in PT mode at 4 mM iron. B) Heating capacity (SLP in W.g^-1^) of NCs (black) and NC@TRAIL (red) in PT mode. C) MDA-MB-231-WT and DKO (10% FCS) cell death due to the effect of the TRAIL alone ([TRAIL] = 10 nM), NCs and NC@TRAIL at 37 °C and in PT mode. D) Early and late apoptosis and/or necrosis were determined by annexin V and propidium iodide (PI) staining after PT treatment. For each condition the percentage indicated in each compartment represents: viable cells (left, bottom, annexin V- and PI-), early apoptosis (right, bottom, annexin V+ and PI-), late apoptosis and/or necrosis (left, up, annexin V+ and PI+) and the debris (right, up, annexin V- and PI +).

Photothermal experiments were then performed on the same MDA-MB-231-WT breast cancer cell line and the photothermal efficacy of NC@TRAIL was evaluated by using a NIR laser with short stimulation (8 min.) at 808 nm 62, and at a low power density of 0.3 W.cm^-2^, which produces no non-specific heating without any NCs. Remarkably, for both NCs alone and NC@TRAIL, the temperature increased from 37 to 43 °C for a concentration as low as 4 mM (Figure 3A: NC@TRAIL and NCs, red and black lines, respectively). Under MHT, to reach the same temperature, a concentration at 12 mM is required. As a result, the SLPs calculated for this condition are very high, with values of (920 ± 34) W.g^-1^ and (740 ± 74) W.g^-1^, for NCs and NC@TRAIL, respectively (Figure 3B). In the PT mode, the SLP value of NC@TRAIL is, therefore, almost 10-fold higher than that for MHT (98 ± 3) W.g^‑1^, making PT more advantageous than MHT for thermal therapy with this nanovector.

TRAIL alone, NCs and NC@TRAIL, with or without NIR stimulation (8 min.) at 808 nm, were then compared with controls at 37 °C (Figure 3C). In parental cells, and in the absence of PT the death rate was 43% for NC@TRAIL and 20% for TRAIL alone, while NCs display almost no non-specific toxicity (Figure 3C). PT, *per se*, does not alter the viability of MDA-MB-231-WT cells with TRAIL or NCs, whereas with NC@TRAIL it raises the death rate from 43% to 90% (Figure 3C, PT). Thus NC-mediated hyperthermia produced by laser irradiation selectively increases TRAIL-induced cell death more than MHT, and at a lower iron concentration.

Under PT conditions NC@TRAIL did not cause cell death in TRAIL-receptor-deficient MDA-MB-231-DKO cells (Figure 3C, DKO), which establishes that the above effect is TRAIL-selective. At a higher iron concentration (12 mM, Figure S4A-C), PT is effective against DKO cells (~70% cell death at 48 °C). In this case, since the receptor is not bound to the target cells, the increase in the death rate can only be due to the proximity of NC@TRAIL to the target, which increases the temperature globally, leading to cell damage typical of hyperthermia.

To confirm these results and to differentiate between the different cell death mechanisms, flow cytometry was performed by using annexin V and propidium iodide staining under different conditions: control, TRAIL alone, NCs alone and NC@TRAIL at 37 °C (Figure S5) and after NIR stimulation in MDA-MB-231-WT cells (Figure 3D). The percentages of apoptosis and necrosis were determined for each case. Whereas TRAIL alone induced 27 ± 5% apoptosis in MDA-MB-231-WT, it rose to 40 ± 7% for NC@TRAIL (Figure S5). After the short NIR stimulation at 808 nm, the cell death rate in the presence of TRAIL alone was unchanged compared to the 37 °C control condition. Under PT conditions, NCs alone induce 15% apoptosis whereas with NC@TRAIL the cell death rate was 73 ± 12%. A control experiment, performed by mixing (no grafting) NCs and TRAIL before PT treatment (Figure S5), resulted in 27 ± 4% apoptosis, only slightly increased by PT (32 ± 5%). This demonstrates that the synergistic effect experienced with NC@TRAIL is not simply due to a sensitization of the cells by TRAIL ligation, and that TRAIL and NCs must be physically connected to provide the massive cell death experienced under PT. To summarize, flow cytometry results (Figure 3D) are in agreement with cell death shown in Figure 3C.

To further characterize the effect of NC-mediated thermal treatment, mere incubation of MDA-MB-231-DKO cells in water baths at 45, 55, 60 and 70 °C was performed for the same duration (8 min.). This also induced non-specific cell killing (Figure S6). However, under these conditions, the cells survived the short thermal incubation at 45 °C. To reach significant cell death, a temperature over 50 °C was needed, with about 55% cell death when incubated at 55 °C, and more than 80% at higher temperatures (60 and 70 °C). This temperature range (55-70 °C) belongs to the category “injury treatments” which is known to cause severe damage. It is noteworthy that cell death was not observed for any system at 45 °C, which is higher than that reached in the laser experiments at 4 mM iron (42-43 °C). This result is in agreement with the fact that for NCs alone, only minor cell death was achieved in the PT mode. In the case of NC@TRAIL, however, the PT-mode cell death rate for WT cells (in the 80% range) suggests that another mechanism is implicated, in addition to TRAIL-induced cell death, which is not caused by the mere elevation of temperature. In previous work 63, it was demonstrated that MHT is more efficient in inducing cell death *in vitro* than a water bath, suggesting that cell damage must be due to temperature-dependent alterations in molecular pathways.

NC@TRAIL-induced cell killing proceeds through apoptosis via engagement of TRAIL receptors 56 both at 37 °C and during moderate hyperthermia (incubation in a water bath 52, MHT or PT). How moderate hyperthermia under PT conditions enhances TRAIL-induced apoptosis remains unknown. One possibility is that PT creates hotspots where the temperature is locally increased for a short period. There are reports that under MHT conditions 64 the local temperature at the NP surface might be different from that of its surroundings, even if no macroscopic temperature changes are detected. Significant local heating can be produced very close to the magnetic NP surface when iron oxide NPs are exposed to an AMF 65, which enables specific pathways such as the activation of ion channels and neurons 65.

**Figure 4:**
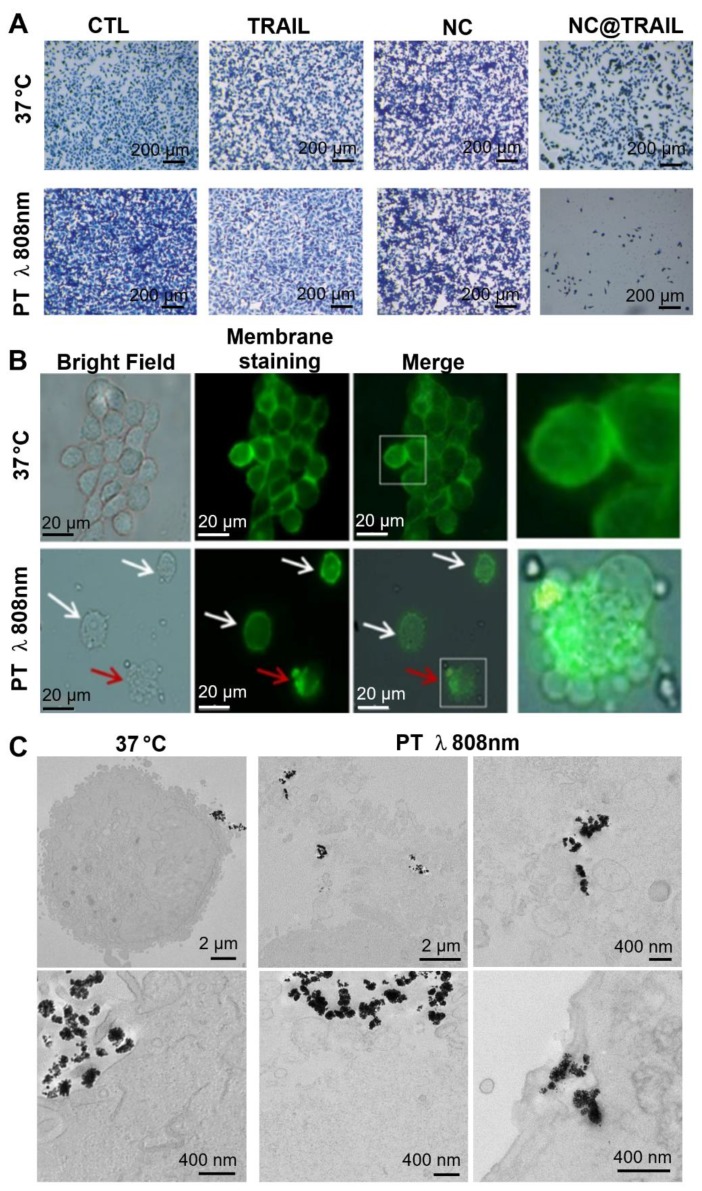
A) Optical microscopy Images of the different systems used (control, TRAIL alone, NCs and NC@TRAIL under different conditions: [TRAIL] = 10 nM, [Fe] = 4 mM, 16 h incubation at 37 °C and laser treatment at 808 nm (0.3 W.cm^-2^) for 8 min. MDA-MB-231-WT cell death determined by methylene blue staining (blue indicates viability, grey-white indicates cell death). B) Bright field microscopy and Green Mask membrane staining of MDA-MB-231-WT cells, showing the effect of NC@TRAIL under PT at 808 nm (0.3 W.cm^-2^). White arrows show the fragility of the cell membrane as compared to the well defined membrane of untreated cells; red arrows show the disruption of the lysosomal membrane caused by NC@TRAIL attached to its agonist death receptor, leading to cell death. C) TEM imaging after treatment (control on the left, PT treatment on the right). Whereas in the control the membrane is well delimited, after PT treatment membranes in the vicinity of NC@TRAIL are highly disrupted. Membrane penetration by NC@TRAIL was also observed (bottom right).

Recent work described a new principle for controlling NP rotation and inducing apoptosis via mechanical forces exerted on membranes by targeted NPs (nanovectors) submitted to a dynamic magnetic field 66. In the present study, the results for NC@TRAIL suggest that the presence of hotspots in the vicinity of the NPs at the cell surface could increase the thermal fluctuations of the particles and thus damage the membranes by a similar mechanism 66. Nevertheless, at this stage it is not possible to differentiate between a purely thermal effect and a mechanical effect induced by local heating by the laser. Figure 4A shows optical microscopy images of methylene-blue-stained cells of the different systems before and after laser treatment. Fluorescence microscopy images (Figure 4B) revealed that after laser irradiation the membranes of cells incubated with NC@TRAIL were severely damaged and the lysosomal membrane was disrupted. Transmission electron microscopy (TEM) provided further evidence for cell membrane disruption by NC@TRAIL. Figure 4C shows a clear difference between the control at 37 °C (left), where the cell membrane is well delimited, and that after photothermal treatment (PT) at 808 nm (right), where the membrane is highly disrupted in the vicinity of NC@TRAIL. Figure 4C (PT, bottom right) shows also the penetration of NC@TRAIL after PT treatment, without internalization within endosomes. Other TEM images (Figure S7) evidence that NC@TRAIL is only bound at the cell surface, with none internalized at the end of the PT treatment.

Therefore, whether the damage is caused by hotspots or mechanical forces due to the presence of the nanovector and its receptor at the cell membrane, the result converges towards a localized cell death. This result was not observed in DKO cells, because of the absence of a TRAIL-specific cell receptor, which excludes any mechanism based on the interaction of NC@TRAIL with its receptor.

**Figure 5:**
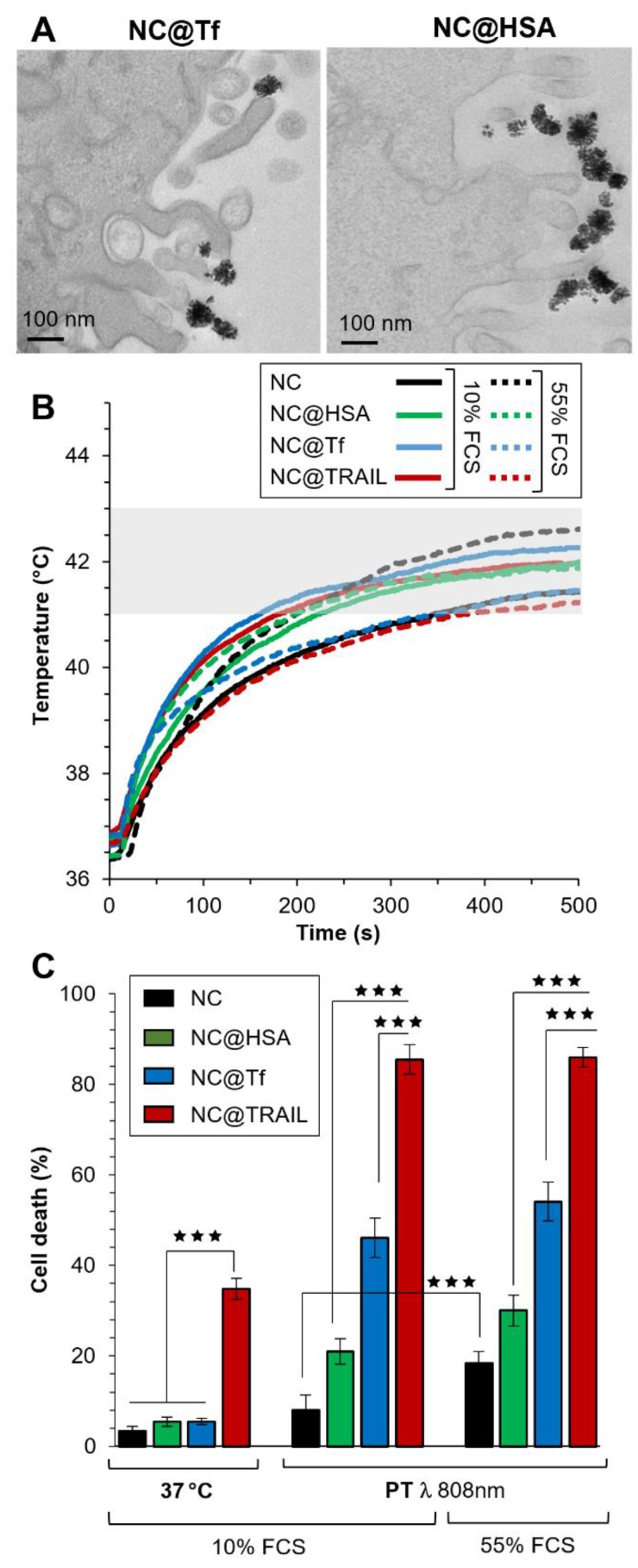
A) TEM imaging of NC@Tf and NC@HSA at the cell membrane after PT treatment. B) Temperature increment for NC@TRAIL (red), NC@HSA (blue) and NC@Tf (green) with MDA-MB-231-WT cells in PT mode at 4 mM iron in presence of 10% and 55% FCS. C) MDA-MB-231-WT (10% and 55% FCS, respectively) cell death, due to NCs, NC@HSA, NC@Tf and NC@TRAIL after 16 h incubation at 37 °C or in PT mode at 4 mM iron.

To confirm the importance of interactions between the nanovector and the corresponding membrane receptor in our system, the iron oxide NCs were functionalized by other proteins: transferrin (Tf), which is known to be internalized by transferrin-receptor-mediated endocytosis, and human serum albumin (HSA) which could also be internalized to a lesser extent by interaction with a receptor at the cell surface 18. In Figures 5A and S8, TEM images of NC@Tf and NC@HSA show unambiguously that these nanovectors were only bound to the cell membrane, with none internalized at the end of the NIR stimulations.

NC@Tf and NC@HSA were used at the same iron concentration (4 mM) to compare them with NC@TRAIL as regards their ability to trigger MDA-MB-231-WT cell death by PT or at 37 °C. As the concentration used for photothermal treatment was lower with a better efficiency than that used in MHT, PT only was used for the treatment of cells with NC@Tf and NC@HSA. In the same way as for PT mediated by NC@TRAIL, PT mediated by NC@HSA and NC@Tf raised the temperature to the moderate hyperthermia range. PT treatment was performed at two different FCS concentrations, 10% and 55% (Figure 5B), in order to mimic more closely the *in vivo* environment.

Contrary to TRAIL, Tf and HSA alone at 37 °C do not cause cell death (Figure 5C, left). When PT was applied, NC@HSA and NC@Tf induce cell death albeit less than NC@TRAIL (Figure 5C, left). In agreement with recent findings that HSA 18 interacts with fewer receptors than Tf 55, the pro-apoptotic potential of NC@Tf is found to be higher than that of NC@HSA 18. The cell death rate induced by PT appears thus to be related to the number of receptors at the cell surface for each protein. Both the transferrin 67-70 and the TRAIL receptors are overexpressed in cancer cells 49, 50.

To further investigate the implication of the interaction between the specific receptors at the cell membrane and the nanovectors in the PT-induced death mechanism, MDA-MB-231-DKO cells were incubated with NC@TRAIL, NC@HSA and NC@Tf, treated under the same PT conditions, and analyzed by flow cytometry (Figure S9). NC@HSA and NC@TF induce 22 ± 4% and 28 ± 5% of cell death, respectively, much more than NC@TRAIL, 9 ± 2%.

This demonstrates that the selectivity of the interaction between a nanovector and its specific receptor at the cell membrane plays a major role in inducing cell death in the PT mode.

Even in the more complex medium with a high FCS concentration (55%), there was a small enhancement of the cell death rate for NC@Tf and NC@HSA. The killing efficacy of NC@TRAIL with PT is not altered in the protein-rich environment, the death rate under these conditions being much the same whether the cells were stimulated in 10% FCS or in 55% FCS, which mimics more closely the *in vivo* situation (Figure 5C). This indicates that the binding of functionalized TRAIL to its receptors, expressed by the target cells, is unlikely to be compromised by the tumor microenvironment.

To summarize, these results demonstrate that a therapeutic effect is reached at lower doses of nanovectors for PT than MHT, in the mM range. In pre-clinical testing, such a concentration could be reached in the tumor if only 2% of the injected dose ends up within the tumoral mass. Indeed, by injecting a dose typically used *in vivo* for iron oxide nanoparticles (e.g. 200 µL at 3 g of Fe/L 27), for a tumor 5 mm in diameter (65 mm^3^), and with 2% of the dose reaching the tumor, the intra-tumoral concentration would be [Fe]=3.3 mM, which is very close to that of 4 mM where the combined PT and TRAIL treatment is very efficient.

Taken together, these results establish the remarkable efficiency of PT and MHT mediated by NC@TRAIL, and a synergy between the effects of moderate hyperthermia and TRAIL itself. They reflect a mechanism where there is direct contact between the vectorized NCs and its receptor during the thermal treatment. Cell death induced by NC@Tf and NC@HSA under these conditions is necessarily associated with the proximity of the NCs to the membrane. Regardless of whether MHT or PT is used with these iron-based vectors, cell death is caused merely through disruption of the plasma membrane due to hotspots in the vicinity of the nanovector. This hypothesis is confirmed by TEM which evidences the presence of the different nanovectors (NC@TRAIL, NC@Tf and NC@HSA) at the cell membrane just after PT treatment. These results thus provide the basis for novel and promising NP-mediated thermal therapies in oncology. PT appears to be the most effective approach, as it requires lower iron oxide concentrations to produce an elevation of temperature sufficient to cause cell death.

## Conclusions

This work demonstrates that the nanovector NC@TRAIL is an excellent magnetothermal and photothermal nanoheater. The apoptotic effect of TRAIL combined with the heating effect of iron oxide nanoclusters was studied in MDA-MB-231-WT cells resistant to TRAIL and in a complex medium (up to 55% FCS). Here we show that NC@TRAIL induces cancer cell death by the intimate interaction of the NC with its TRAIL receptor during the thermal treatment. It implies hotspot generation around the nanoclusters and, therefore, at the cell surface in the vicinity of the targeted receptors, leading to disruption of the membrane and subsequent cell death. We have confirmed this mechanism by using nanovectors grafted with other proteins, transferrin and HSA, and showing that at the same iron concentration and temperature increment, the cell death rate is related to the attachment at the cell surface. TEM images are consistent with this hypothesis, revealing massive membrane damage after PT treatment, and flow cytometry reveals that the cell death mechanism consists of a mixture of early and late apoptosis accompanied in some cases by necrosis. We have also shown that cell death is not directly related to the global temperature increment. Moreover, no effect was observed in TRAIL-receptor-deficient DKO cells. For these cells, where the nanovector cannot be targeted, a successful alternative strategy is to use PT in the “suppressive hyperthermia” range (48 °C) by adapting the dose of iron (to 12 mM), and keeping a safe value for the laser power density (0.3 W.cm^-2^).

## Supplementary Material

Supplementary figures.
