# Supplementary material for: TRAIL acts synergistically with iron oxide nanocluster-mediated magneto- and photothermia
Source: Theranostics. 2019 Aug 14;9(20):5924–36. doi: 10.7150/thno.36320 (PMC6735372; doi:10.7150/thno.36320)
Supplement: Supplementary file 1 — Supplementary figures. [file thnov09p5924s1.pdf]

Supporting Information

TRAIL acts synergistically with iron oxide nanoclusters-mediated magneto- and photothermia

Hanene Belkahla[a,b,c], Eva Mazarío[a], Anouchka Plan Sangnier[d], John S. Lomas [a], Tijani Gharbi[b], Souad Ammar[a], Olivier Micheau[c]‡, Claire Wilhelm[d]‡, and Miryana Hémadi[a]‡

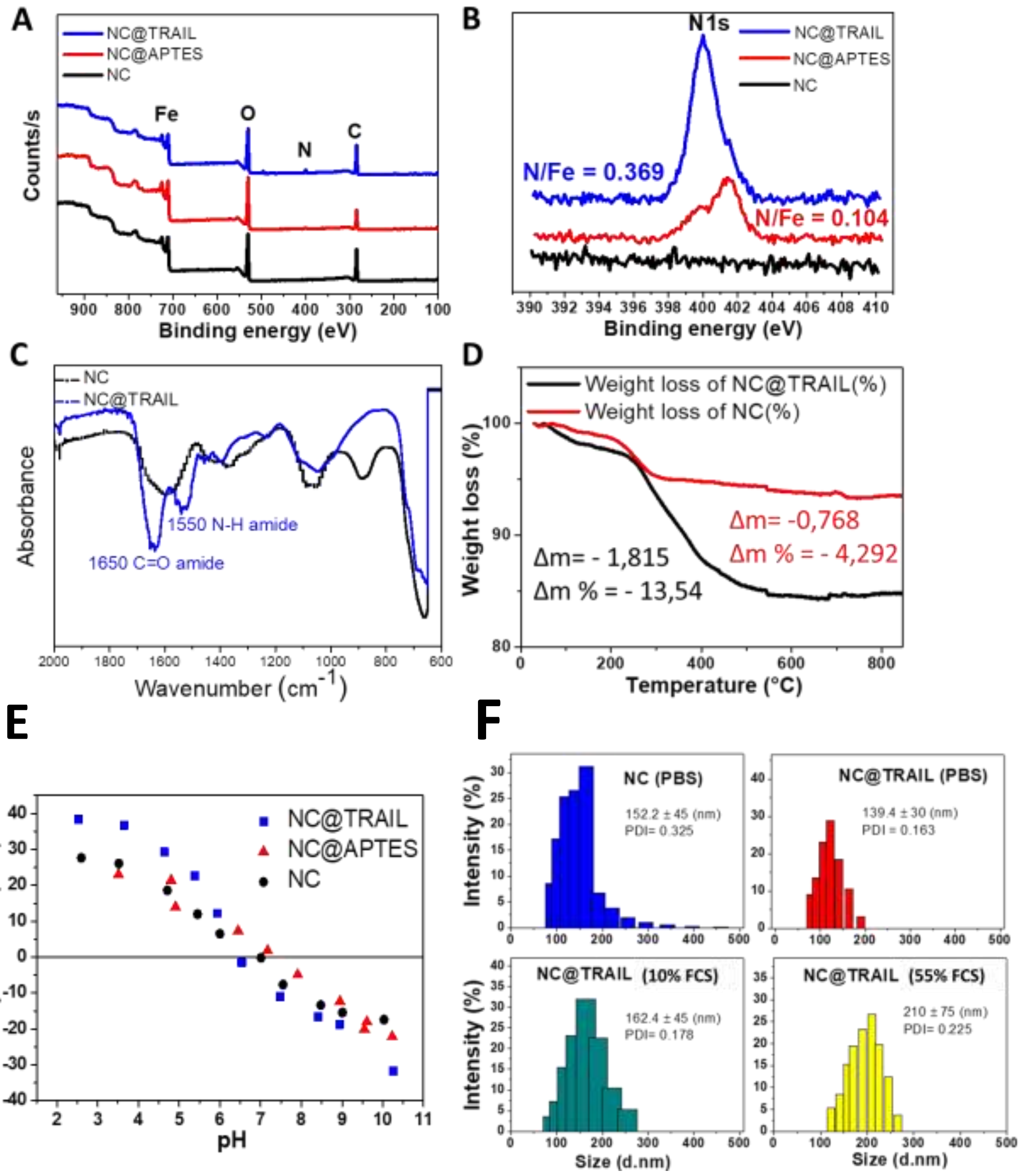

G

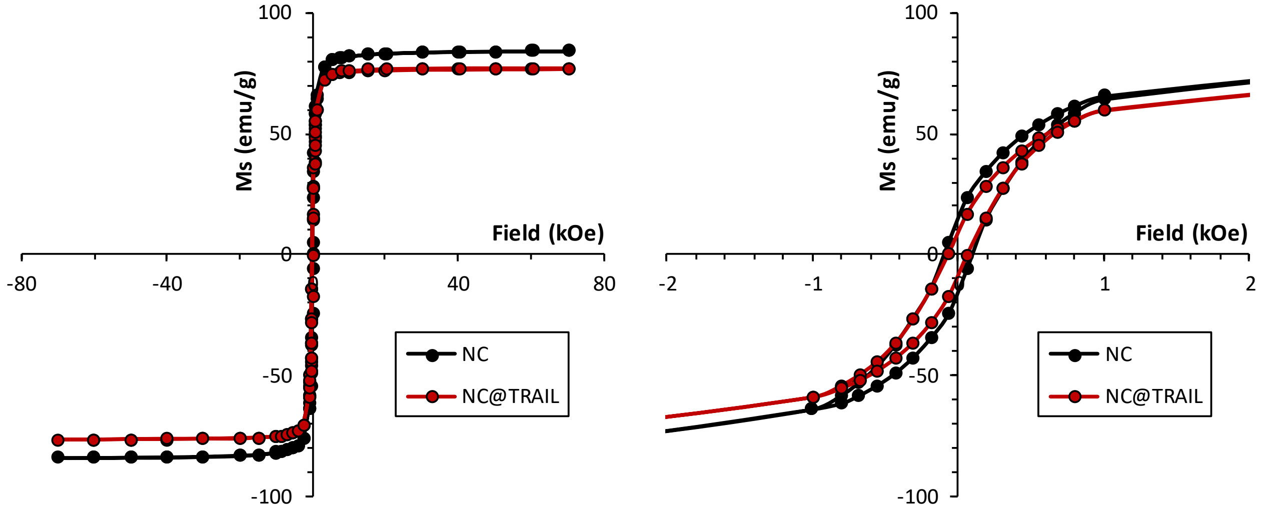

Figure S1: **A**. X-ray photoelectron spectroscopy survey spectra of NC, NC@APTES & NC@TRAIL **B**. The high-resolution N1s spectra of NC, NC@APTES & NC@TRAIL **C.** FTIR spectra of NC and NC@TRAIL**, D**. Thermogravimetric analysis of NC and NC@TRAIL, **E**. Zeta potential measured as a function of pH on aqueous suspension of NC@APTES and NC@TRAIL **F**. DLS profile of NC@TRAIL in PBS, 10% and 55% FCS. **G.** Magnetization curves at 5K for NC and NC@TRAIL.

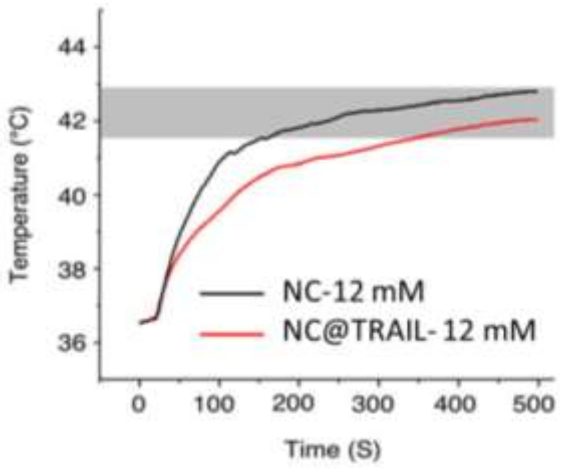

Figure S2: Plot of temperature versus time for NC and NC@TRAIL induced by MHT (471 kHz, 180 G) of MDA-MB-231-DKO cells incubated in 10% FCS with NC and NC@TRAIL at [Fe]=12 mM.

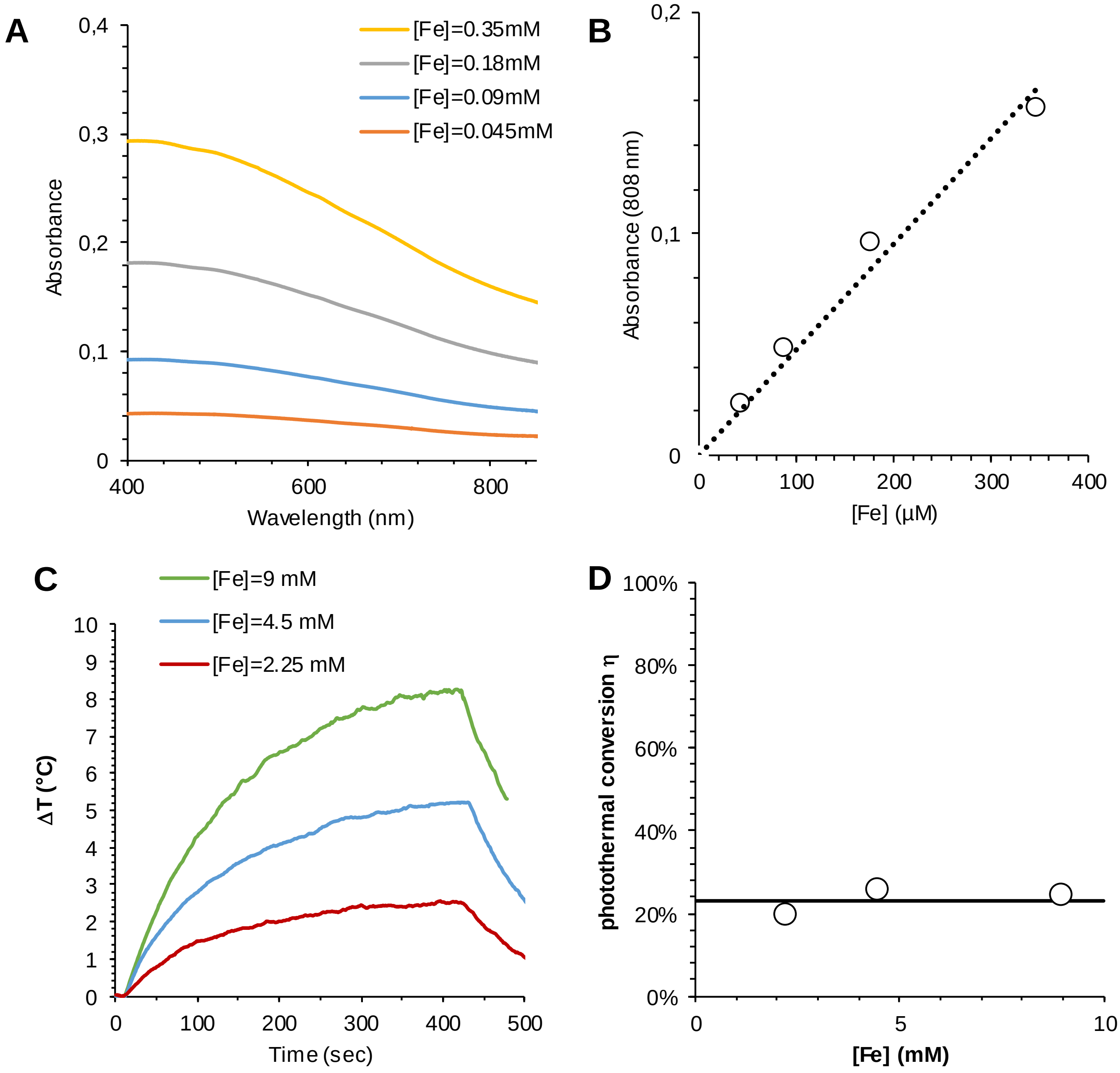

Figure S3: **Determination of the light-to heat photothermal efficiency conversion parameter η.** **A**. Absorbance (A) spectra at different concentrations of the nanoclusters ([Fe]). **B**. Plot of the absorbance at 808 nm with respect to iron concentration. The molar extinction coefficient (ε) was determined from these data according to the Beer-Lambert equation: A= ε.l.[Fe] with l the length of the cuvette (1 cm): ε = 475±50 $M^{-1}cm^{-1}$. **C**. Typical curves of the temperature increase ΔT over time showing the temperature evolution during photothermal measurements. Laser was turned on after 10 sec of recording. As soon as the plateau temperature $\Delta T_{plateau}$ was reached, the laser was turned off, and the heat relaxation phase was recorded, in order to calculate the light-to-heat conversion coefficient. **D**. Light-to-heat conversion coefficient (η) extracted from the heating curves shown in C for the three different sample concentrations, according to the formula: $\eta = \frac{\Delta T_{plateau}.m.C.B}{P_0.(1-10^{-A})}$, with $\Delta T_{plateau}$, the final temperature increase; P_0 the incident power of the laser (here 0.12 W, corresponding to a power density of 0.3 W/cm^2 and a sample surface area of 0.4 cm^2); A the absorbance of the sample (provided by the ε value); m the mass of the sample, C the specific heat capacity (4185 J/g/K), and B the constant rate of heat dissipation. B was calculated from the

relaxation phase as: $e^{-Bt} = \frac{\Delta T(t)}{\Delta T_{plateau}}$, leading to B=0.01 ± 0.002 s^{-1}. This led to an average η value of 23±3%.

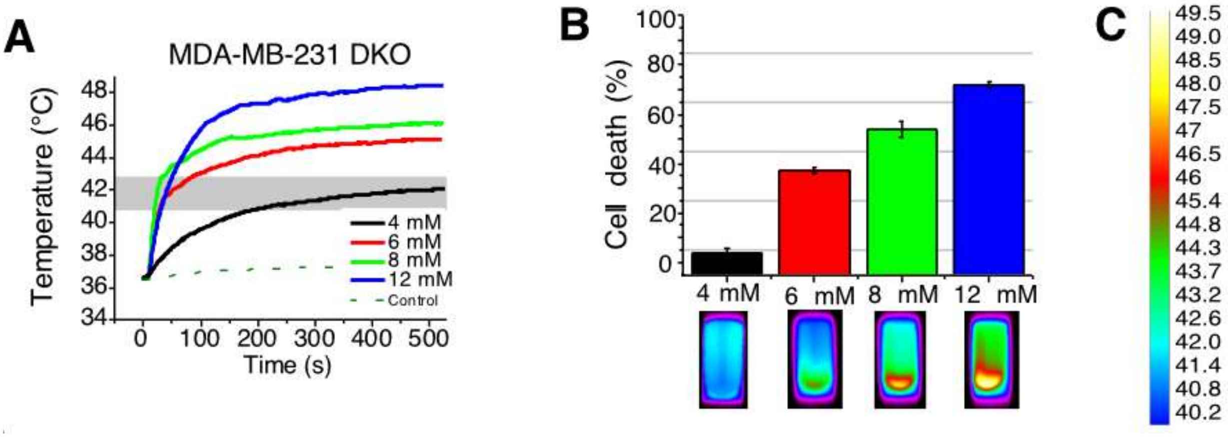

Figure S4: A) PT-mode temperature increment for NC@TRAIL in MDA-MB-231-DKO cells with different iron concentrations. B) MDA-MB-231-DKO cell death in PT mode with different iron concentrations. C) Temperature scale bar.

MDA-MB-231 WT

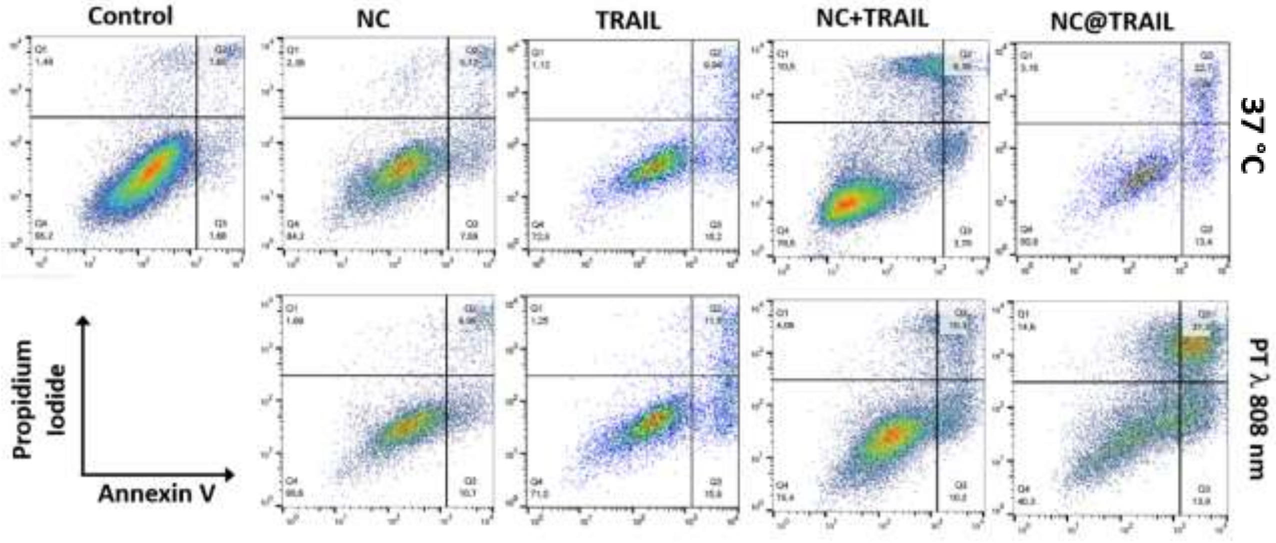

Figure S5: Early and late apoptosis and/or necrosis were determined by annexin V and propidium iodide (PI) staining of MDA-MB-231-WT treated by NC, TRAIL, NC+TRAIL and NC@TRAIL at 37 °C and after PT treatment. Total cell death is given in bold as a percentage.

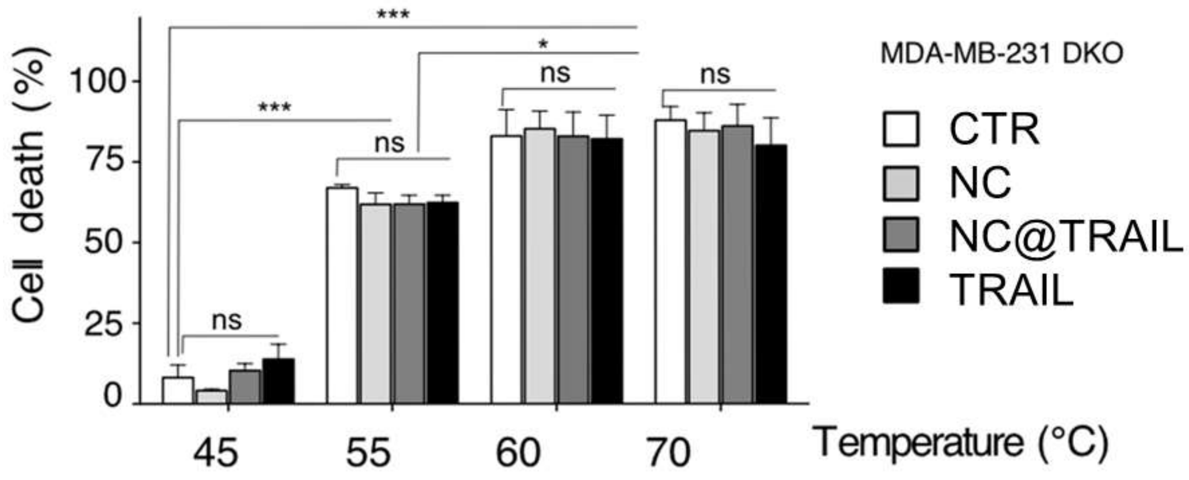

Figure S6: MDA-MB-231-DKO cell death after incubation with TRAIL alone, NC and NC@TRAIL ([Fe]=4 mM) at different temperatures: 45, 55, 60 and 70 °C.

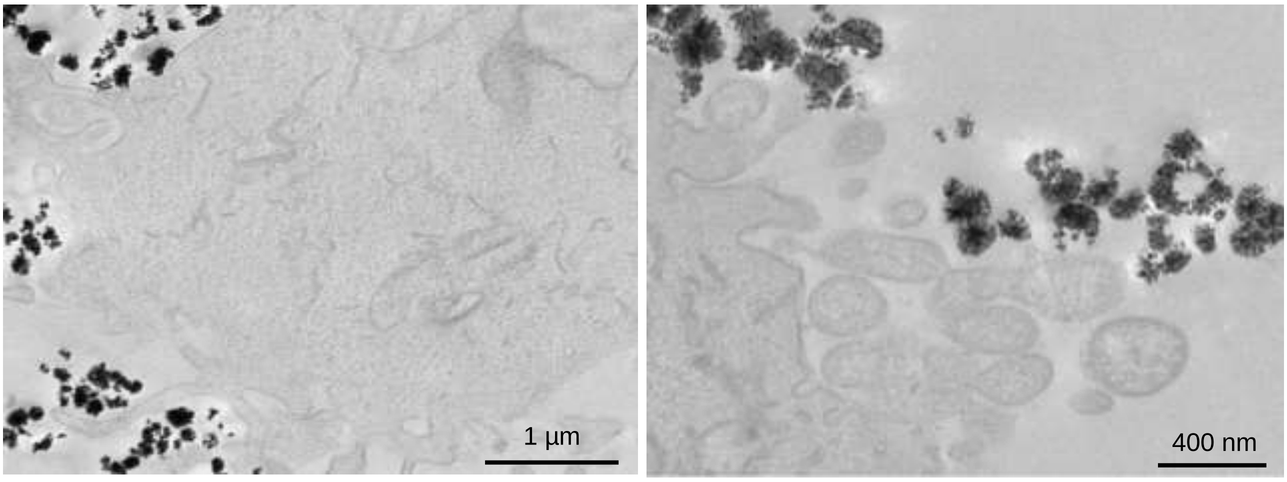

Figure S7: TEM images of MDA-MB-231 cells incubated with NC@TRAIL at [Fe]= 4 mM, immediately after the incubation. Nanoclusters are all located at the cell membranes.

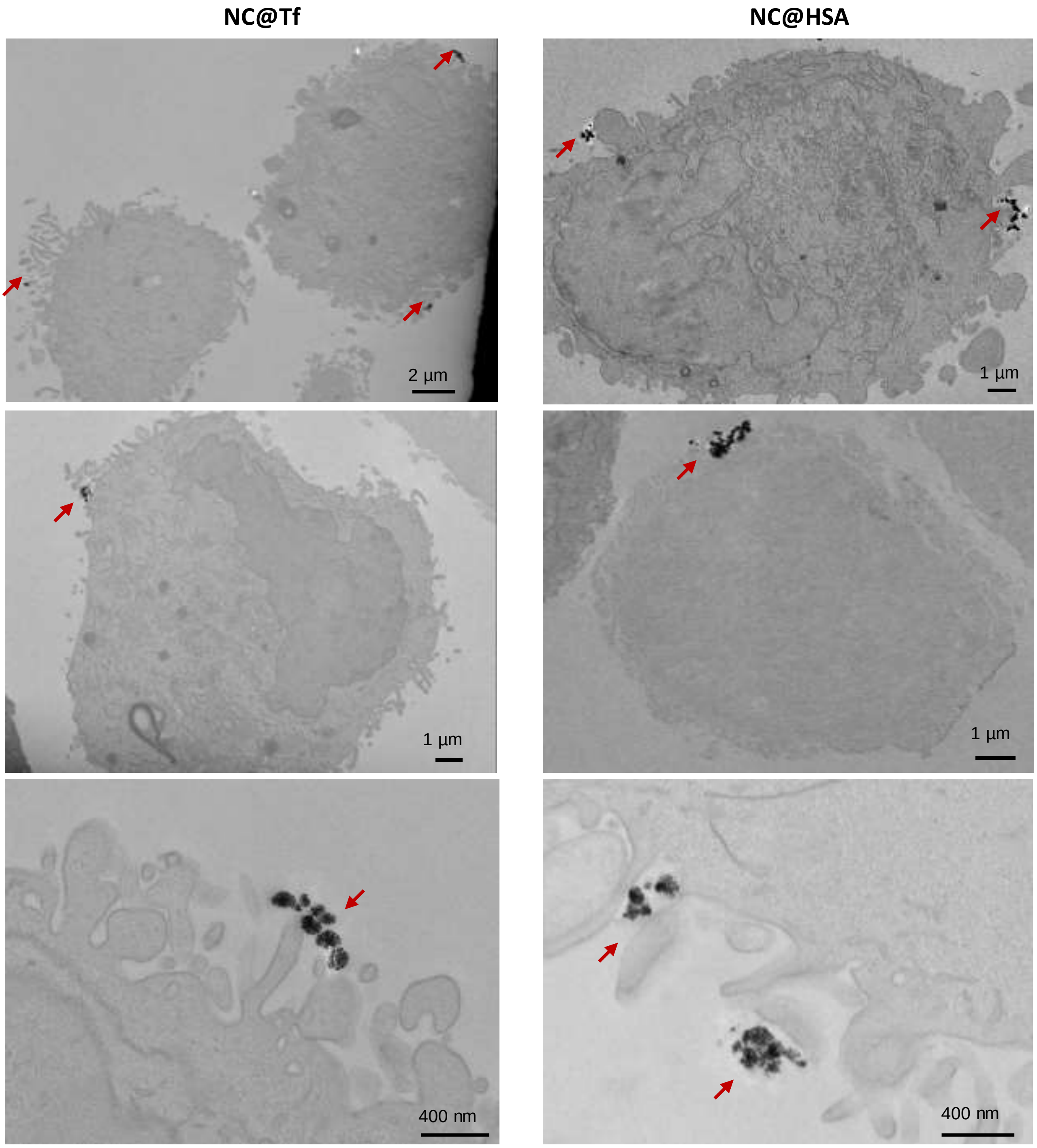

Figure S8: TEM images of MDA-MB-231 cells incubated with NC@Tf (left) and NC@HSA (right) at [Fe]= 4 mM, immediately after the PT treatment. Nanoclusters are all located at the cell membranes, with none internalized, evidencing local membrane-delimited action.

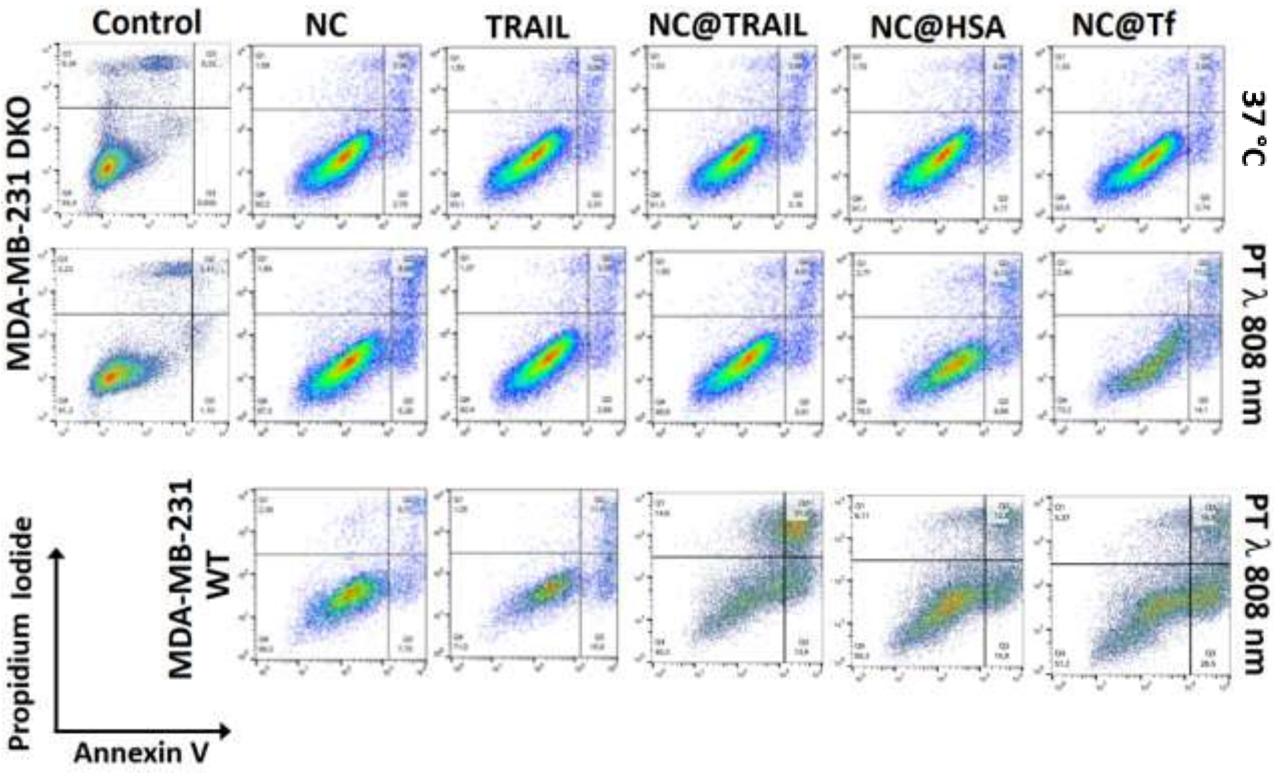

Figure S9: Early and late apoptosis and/or necrosis were determined by annexin V and propidium iodide (PI) staining of MDA-MB-231-DKO treated by NC, TRAIL, NC@Tf, NC@HSA and NC@TRAIL at 37 °C and after PT treatment, and their comparison with MDA-MB-231-WT results after PT mode. Cell death is given in bold as a percentage for each measurement.

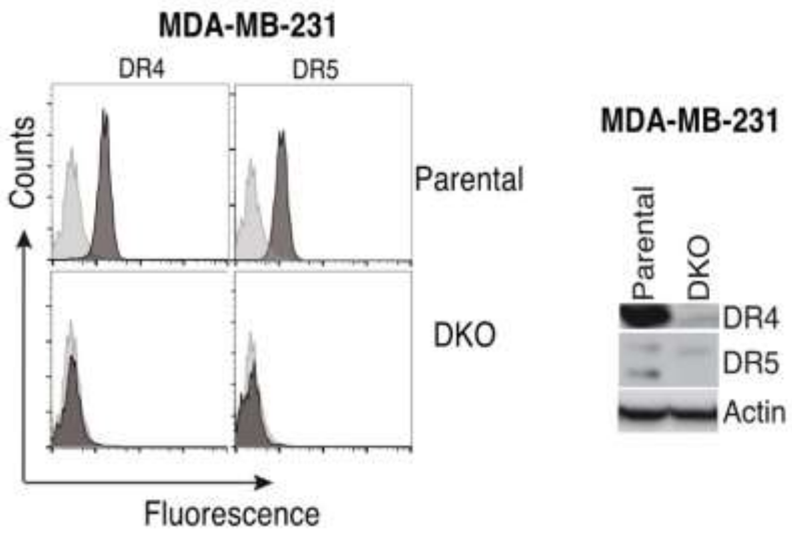

Figure S10: Left: Identification by flow cytometry of the presence of TRAIL receptors DR4 and DR5 in parental cell line (WT) and TRAIL-deficient one (DKO). Right: TRAIL-DR4 and TRAIL-DR5 immunoblots obtained from parental (WT) and DKO cell lysates.
